# Co-creation of a Serious Game About Radiotherapy: Participatory Action Research Study With Children Treated for Cancer

**DOI:** 10.2196/34476

**Published:** 2022-05-31

**Authors:** Catarina Cederved, Jon Back, Charlotte Ångström-Brännström, Gustaf Ljungman, Gunn Engvall

**Affiliations:** 1 Department of Women's and Children's Health Uppsala University Uppsala Sweden; 2 Department of Informatics and Media Uppsala University Uppsala Sweden; 3 Department of Nursing Umeå University Umeå Sweden

**Keywords:** children, participatory action research, game design, radiotherapy, education, supportive care, oncology

## Abstract

**Background:**

Children with cancer who have to undergo radiotherapy can experience fear, because they have no prior knowledge of the treatment. One way of teaching children about the treatment and reducing their fear is to prepare them for it through serious games. Involvement of the end user in the design process within medicine is a way of ensuring that the product being developed will fit the intended user.

**Objective:**

The aim was to outline the contributions made by children and their parents through participatory action research when designing a serious game about radiotherapy.

**Methods:**

By means of participatory action research, children and their parents participated in the development of a serious game about radiotherapy. Nine children (7-10 years old) were included, each with an accompanying parent. A qualitative approach was used that included interviews and participant observation. Six rounds of iterative development process were used with the children and their parents. Meetings with the children were held either face-to-face or online. Each round resulted in a list of suggestions for changes to the game. A thematic analysis was performed based on the list of proposed changes, underpinned by all gathered data, to highlight how the children’s participation changed the game.

**Results:**

Two main themes were identified. The first theme was “The children’s participation was affected by their health and treatment” and included the following subthemes: “an opportunity to share emotions and perceptions of radiotherapy” and “the possibility to participate was affected by the severity of the disease.” The second theme was “participation allowed becoming an active part of game development” and included the following subthemes: “the opportunity to express sentiments about the game,” “the emergence of a playable game through the children’s contributions,” and “the necessity of understanding the text.”

**Conclusions:**

The method used in this study made the children active participants, and our results suggest that this method can be used by health care researchers to cocreate serious games with children. It is necessary to inform the children involved that the process takes time, and that the process can be altered to allow as much participation as possible without placing a burden on them. The children’s illness affected their possibility to take part; thus, it is crucial to accommodate the children’s needs when conducting similar studies. The parents’ participation facilitated the meetings for their children, even though their involvement in the game design was negligible.

## Introduction

Children with cancer face many challenges and distressing events [1]. The procedures that the children have to undergo when receiving treatment are previously unknown to them and often cause fear [2,3]. Fear of the unknown has been described as a fundamental fear, and in situations with unknown elements, patients should be provided with tools to increase their ability to cope [4]. Radiotherapy (RT) is one of the major treatment modalities that can cure or alleviate cancer, depending on the cancer diagnosis [5]. For children, it can be difficult to receive RT since they must remain in a fixed position without moving, and they are left in the treatment room by themselves [6]. Some children need daily anesthesia to cope with the procedure, lasting for several weeks and accompanied by fasting periods; this is suboptimal for a growing body [6]. It has been suggested that with the right preparation children may have a greater chance of coping with the RT procedure, allowing them to receive the treatment without sedation [7-9].

The current project is based on previous research conducted by Engvall et al [10], who developed a digital story describing the RT procedure that was delivered as an application on a tablet designed for children. The participating children suggested that the application could have been more interactive if it had been designed as a game [10]. When designing serious games such as these, there are several aspects that need to be considered, such as the purpose of the game, the end users, the stakeholders in the project, and how the game can engage the players [11-13]. Serious games are a way to learn and are meant to solve real world problems; in the best case, they are enjoyable and entertaining [14,15]. Involving the end user in development has become common practice when developing games and interactive applications for children in medical facilities [16,17], as this is considered to increase the chances of creating a successful game [18]. Participatory action research (PAR) involves children as cocreators in the research process, allowing them to contribute and take part at different levels of the study [19]. Thus, PAR can strengthen study findings and the interventions being researched [20]. To our knowledge, no serious games have yet been developed to prepare children for the RT procedure. Further, researchers of serious game design have highlighted the significance of describing and reporting the involvement of end users in the design process and in research during the design process [21]. This paper is not primarily meant to point out the relevance of participatory design. Rather, it is meant to show how PAR can be used to bring about changes, using RT as an example. We wanted to create a serious game about RT in collaboration with children with experience of RT, to promote knowledge of the procedure in children who will receive RT in the future. The aim was to outline the contributions made by children and their parents through PAR when designing a serious game about RT.

## Methods

### Study Design

The present study describes the development of a serious game for RT as part of a larger project to reduce RT-related anxiety. The development of the game used PAR by involving children that had undergone RT, their parents, an expert team of health care professionals, game designers, and a research team. A qualitative approach was used that included interviews and participant observation.

### Recruitment of Participants

A nurse at a pediatric oncology center contacted parents with a child who had received or was receiving RT to introduce the study. The inclusion criteria were that the child had received or was receiving RT, was fluent in Swedish, and was between 6 and 15 years old. A total of 14 families were approached, of whom 5 declined to participate. Explanations given for declining included lack of interest in a game project, not having time, and prioritizing school; some families gave no explanation.

### Ethical Approval

Informed and written consent was given by parents on their child’s behalf and by the parents on their own behalf. Written assent was given by the children participating in the study. The study design was approved by the Regional Ethical Review Board in Uppsala, Sweden (2018/264).

### Data Collection

Before each meeting, the parents were contacted with appointment suggestions. Meetings were scheduled at times that most of the children could attend and were held in 2 towns to shorten the traveling distance for the families, meaning the participants were divided into 2 groups. The families were reimbursed for their travel expenses.

The game development was performed as an iterative process [22]. The children met at play meetings where they played and commented on a prototype of the game. The children were observed while playing the game prototype and their gameplay was recorded on a computer. The children were asked questions about elements in the game to further understand how they interpreted them. After the game testing, a semistructured interview with follow-up questions was conducted in a group or individually; these interviews were audiotaped (an interview guide is included in Multimedia Appendix 1).

In the first round, the children and accompanying parents met at the research venue and were offered refreshments. The children discussed their experiences of RT, what games they preferred playing, and talked freely about what they thought should be in the game that was going to be developed. Two investigators were present; one was active and interacted with the children, while the other observed and took notes. In an effort to make the children more comfortable and not feel inferior in the situation, the decision was made to limit the number of adults by not having the design team present. This also solved a logistical problem: the design team was located in a city several hours away from where the study was conducted. In the second round, the children played a prototype of the game in groups of 2 or individually. After the second iteration, a change was made due to COVID-19 to minimize potential infection. The families were given options to meet with one investigator and one other family, meet with only the investigator, or to have a virtual meeting with one investigator over the internet. However, the rounds still had the same structure: the children played the game prototype, sometimes showed their parents the game, and talked about it with their parents if they wanted to. The parents were asked in the later rounds to provide their thoughts and experiences of the game.

### Analysis and Game Design Process

**Figure 1:**
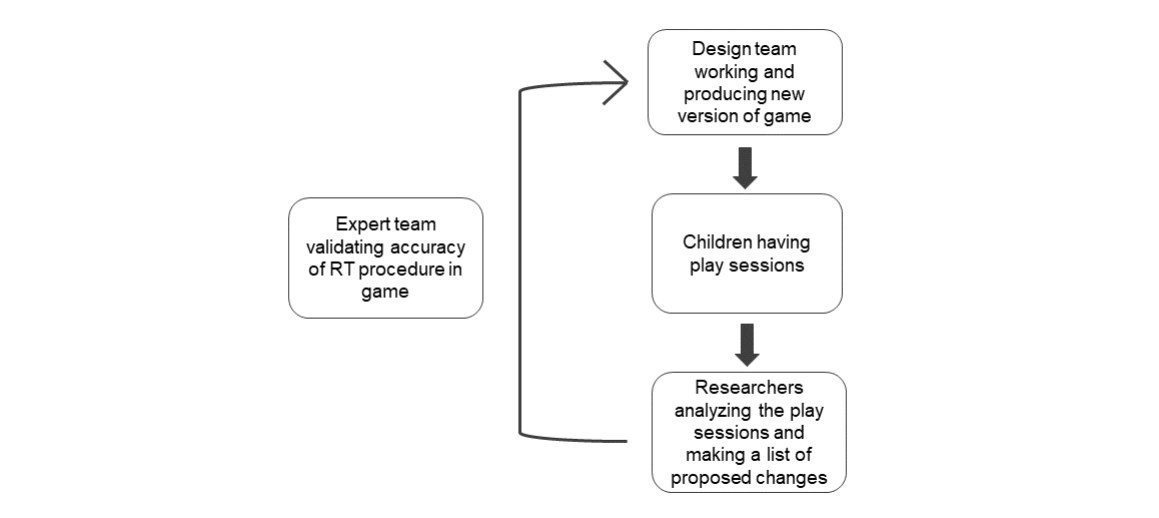
The iteration process. RT: radiotherapy.

The material analyzed from each round with the children and their accompanying parents consisted of 1) screen recordings of gameplay, 2) observational field notes taken during gameplay, 3) summary notes made after each round, and 4) audio recordings of the subsequent interviews. The screen recordings were viewed multiple times; inductive coding was performed immediately after each round. In this way, 3 main coding categories emerged. These were audiovisual cues, game mechanics, and narrative. Therefore, coding of the early rounds informed later rounds. The material from later rounds was coded into the discovered categories. A similar approach was then used to identify codes for the field notes, summary notes, and interviews. All 4 data sets were compared to find commonalities between the children’s play. Codes were also grouped, revealing design suggestions. The suggestions were then prioritized into a list of proposed changes that was presented to the design team within 1 to 2 weeks after each round. After discussions on feasibility and time, the design team made as many of the changes as possible within the given timeframe. The new changes to the game prototype were observed at the next iteration (Figure 1). The iterative procedure with the children was repeated 6 times over a period of 8 months during 2020. None of the children took part in the analysis or the processing of the material.

A thematic analysis based on the content from the list of proposed changes underpinned by all the gathered data was later performed to capture the children’s and parents’ participation in changes made to the game. Data were analyzed through a thematic analysis inspired by Braun and Clark [23]. Codes were identified and grouped and themes and subthemes were formulated, discussed with coauthors, and revised when necessary until consensus was obtained. Finally, quotations and figures were added to illustrate the content.

### Description of the Game

**Figure 2:**
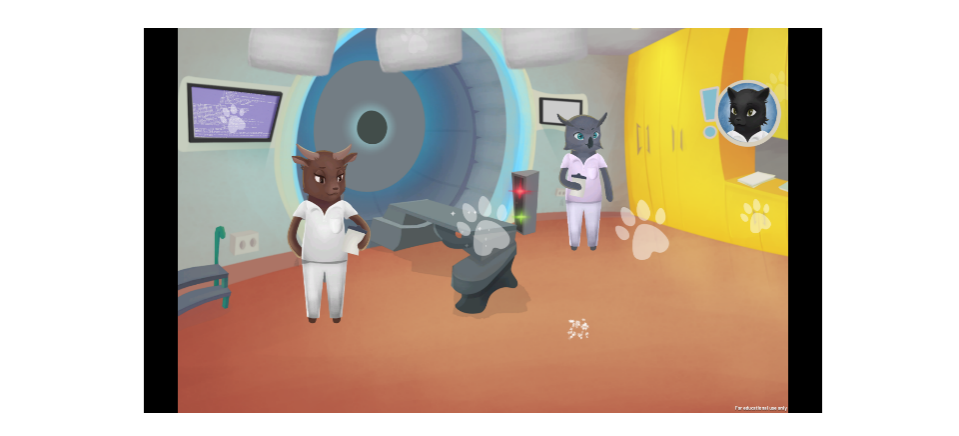
Radiotherapy room after changes were made based on the children’s and parents’ contributions.

**Figure 3:**
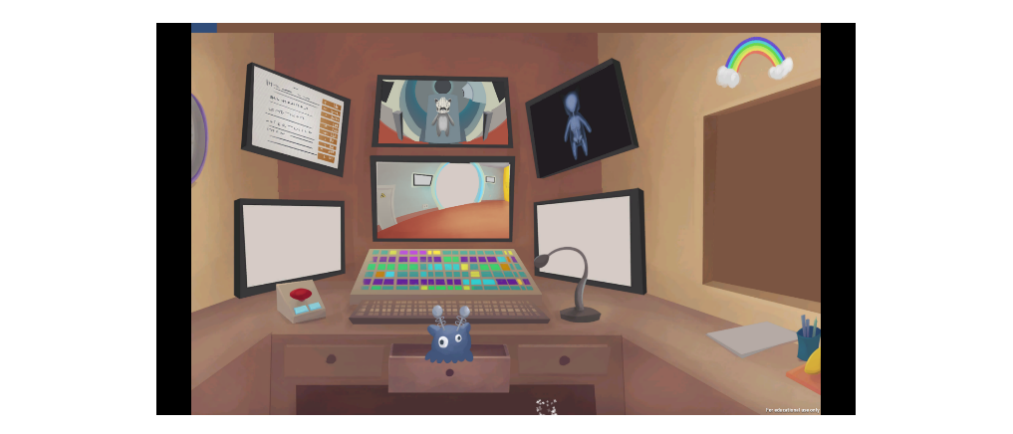
Monitoring room after changes were made based on the children’s and parents’ contributions.

The design team that was assembled to build the game consisted of students from the Department of Game Design at the University of Uppsala and a lecturer who provided supervision. The first version of the game prototype came about after a workshop with the expert team, research team, and design team identified problems, chose the technological platform, and identified the end users. The workshop was held at the clinic that was later portrayed in the game. The first version was a proof of concept developed for children 9 to 15 years old. The game used a third person perspective and was set in a cartoon-style RT clinic where the player followed an avatar through the RT process. The game consisted of linear puzzle rooms in which the player had to complete certain tasks in order to unlock subsequent rooms and tasks. The first version included rooms depicting an RT room, a monitoring room, and a bedroom, as well as mini games not related to the main puzzle. The game was played as a linear loop. The final version of the game came about after the sixth round with the participants and included rooms depicting a reception area, an RT room, a monitoring room (Figure 2 and Figure 3), a narcosis room, a practice room for RT, a kitchen, and a bedroom, which included hidden scenes of a wardrobe and an outdoor environment. The need to complete tasks to be able to progress within the game had been removed, and the game was now played as a “doll-house” style game [24]. The rooms were connected through a game map and all were already accessible from the beginning of play. Every room or environment depicted in the game actually existed at the hospital, apart from the practice room. Some of the mini games corresponded to the RT procedure, while others did not. Some components not relevant to the RT procedure were included in the game to encourage the player to play for a longer time, to encourage engagement with the RT scenes, and to promote understanding of the RT procedure.

## Results

### Description of the Data

Iteration of the game design took place over 6 rounds, each of which included testing with the children, and each of which resulted in a new version of the prototype. Each round included multiple meetings held at different times depending on when the children could attend. The study included 8 children in the first round, and later, 1 additional participant was recruited. Table 1 shows information on how many children participated in each round. All the children had a single parent with them during each round, except for 1 child whose parents took turns accompanying the child. At the time they participated, the children were between 7 and 10 years old and included 1 boy and 8 girls. No child was 9 years old at the start of the project. The children had various diagnoses, including brain tumor, rhabdomyosarcoma, spinal cord cancer, and Ewing sarcoma. Two children chose to leave the study in the fourth round.

Two main themes were revealed by the analysis: (1) the children’s participation was affected by their health and treatment, and (2) participation allowed becoming an active part of game development. Each theme had subthemes that described aspects of the cocreative process.

**Table 1:** Number of participating children in each round and meeting (N=9).

Number of participating children in each meeting, n	Round number					
	Round 1	Round 2	Round 3	Round 4^a^	Round 5	Round 6
Meeting 1	3	4	2	2	1	1
Meeting 2	4	1	2	2	1	1
Meeting 3	0	0	1	1	1^b^	1
Meeting 4	0	0	1^b^	0	1	1^b^
Meeting 5	0	0	1^b^	0	0	1^b^
Meeting 6	0	0	1^b^	0	0	0
Total	7	5	8	5	4	5

^a^Round included newly recruited participant.

^b^Round/meeting was conducted online.

### The Children’s Participation Was Affected by Their Health and Treatment

The children and parents shared how they felt and what it was like when they received RT. Their stories became subthemes: (1) how the meetings provided an opportunity to share emotions and perceptions of RT, and (2) how the possibility to participate was affected by the severity of the disease.

### An Opportunity to Share Emotions and Perceptions of RT

**Figure 4:**
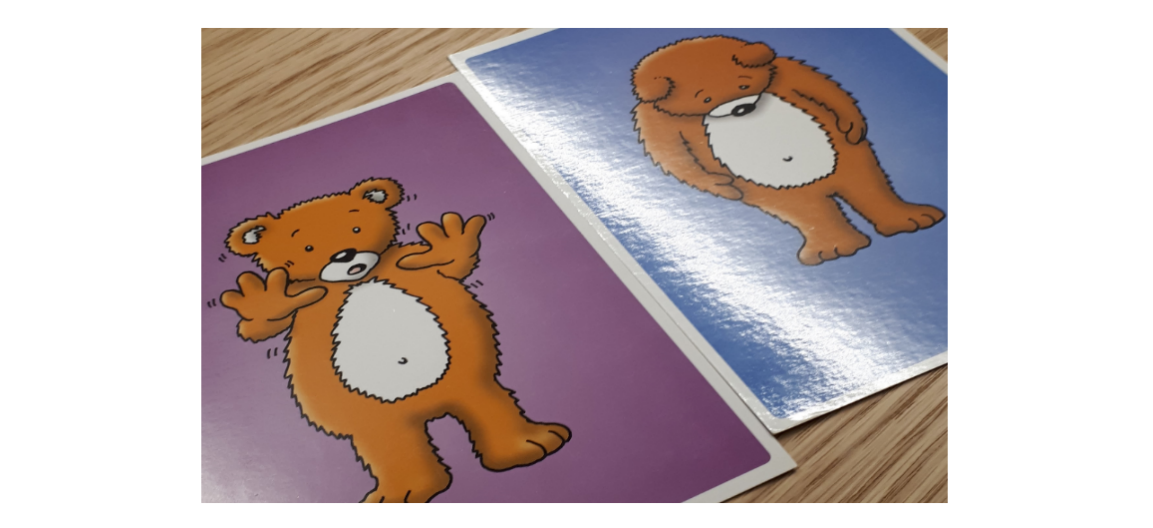
A child’s chosen emotion cards for the emotions the child felt during the first radiotherapy treatment. The cards were printed with the approval of St Luke’s Innovative Resources.

The children described by means of emotion cards how they felt about receiving RT. When talking about RT, a few said that they had been happy, but they did not know how it worked when they received their first treatment. However, most of the children said that they had been worried before receiving RT, because they were not sure what would happen or how it would feel (Figure 4). Hence, the game needed to capture the essence of the RT procedure from a child’s point of view.

One parent expressed that they had felt that their child looked vulnerable due to the number of staff around the child when preparing for RT. After checking with the expert team regarding how many people were usually present, additional personnel were added in the game’s RT room in accordance with the input from the parents.

### The Possibility to Participate Was Affected by the Severity of the Disease

Due to the children’s illness, some of them showed signs of fatigue or lack of strength. Some children had to undergo treatment at the hospital in the morning before coming to a meeting, and some children had to choose between going to school or participating in the study, because they did not have the strength to do both. If they found the testing interesting, they stayed, even though they sometimes showed tiredness. The audio recording of one of the meetings in round 1 with 4 children has audible yawning more than once toward the end. The meeting lasted for a little over an hour. Therefore, the day and length of the meetings were modified and adapted to meet the children’s ability to participate. The families could choose to have the meetings online or could choose a date within a certain week that worked for them.

### Participation Allowed Becoming an Active Part of Game Development

Three subthemes were identified within the main theme: (1) the opportunity to express sentiments about the game; (2) the emergence of a playable game through the children’s contributions; and (3) the necessity of understanding the text. Examples are given below of how the children actively contributed to the game design process.

### The Opportunity to Express Sentiments About the Game

During the first meeting, the children discussed other games they liked to play and explained features they thought should be developed in our game. They reported contrasting gaming habits. Some were used to playing very advanced video games while others hardly played at all. The children agreed that the main purpose of the game was to explain how RT worked, to follow the character through the procedure, and to give the character medication. However, they added that the game should be fun and exciting. Their input led to the first prototype of the game being scrapped due to its long introduction. The game was reconstructed to start with RT. After playing the game, the 10-year-old children and their parents thought that the appearance of the game was similar to the real RT setting, but that the game was boring and childish with too little explanation of how the RT procedure worked. One child said that they wanted a scene that showed what happened when you were sedated for RT. In response to these comments, more information was added to the RT room and an extra scene was added. A narcosis room was also added, as well as a practice room where the player could encounter different elements of RT through comic strips. However, we did not obtain ideas on how to make the game more interesting or amusing. Some of the children who were 7 to 8 years old were under anesthesia during RT, so they could not comment on the accuracy of the portrayal, although they enjoyed the interactions that were in the game for amusement and creative play, especially the mini games. One child said they really enjoyed the game.

The children were shown a map of the game on paper and asked what they thought about it. They gave no opinion as to whether they considered the map good or bad, but when it was implemented in the game, thereby removing the game’s linearity, the children spontaneously used it and understood its function. One of the 10-year-old children was asked in the last round why they returned to the kitchen in the game, even though nothing new had been implemented there, and they answered: “Because it is fun there.” When asked why, they replied, “Because I can pick tomatoes.” Another child, in the 7- to 8-year-old age group, also stated that they thought the game was fun to play because of particular scenes and interactivity. The same child had earlier in the iteration process exclaimed that they thought the game was boring and stupid.

A major feature of the game was teaching the player strategies on how to cope with the RT procedure. However, in the interviews, the children pointed out that one of these coping strategies was incorrectly portrayed. Children that undergo RT are sometimes given a string to hold on to; a parent, in an adjacent room, holds the other end and can pull the string so that the child feels the tug. In the game, the child character was shown holding onto a string, and to visualize the tug, the character’s hand was shown moving. However, the children explained that in fact, they were not allowed to move at all during RT, so the mini game was removed and replaced with a comic strip explaining the strategy.

When discussing what the children did to keep themselves occupied during RT, they said that they often thought about things they would do later that day. One child explained that they thought about food they would like to eat. These accounts from the children led to their specific coping strategies being added to the game’s RT room.

### The Emergence of a Playable Game Through the Children’s Contributions

The children sometimes had trouble with movement in the game, while at other times, they misunderstood the game environment and missed gameplay elements that were intended to give clues on how the game worked. We therefore added a visual aid to indicate the location of items and how to advance the story. These additions allowed the children to play autonomously. Some children had problems using a mouse to control the game, which sometimes irritated them, since it meant that they could not, for example, pick up an item. To facilitate their understanding of how to play and interact with the game, we added an introductory scene as a tutorial, set in a reception area. After reading the tutorial, the children said they understood how to maneuver through the game, but when we asked them how to do it, we realized that they still had some problems. The children’s feedback on the tutorial scene was that it looked like the clinic. Some children asked what they were meant to do when introduced to new scenes during play. The game already featured some scenes with telephones that informed the player what they could do in that scene, so we added them to every scene. Some children found that the sound effects in the game were too loud, especially when playing online with headphones. Therefore, the sound effects were modified and a function was added that let the player turn the music and sound effects off and on and read the text in the game aloud.

Some elements of linearity were kept in the game. For example, the RT procedure in the RT room ran on a loop. The parents questioned this, arguing that the player should not have to sit through the loop before leaving the room. Furthermore, one child in the recordings could be heard saying, “Okay, I’ll play through this part,” only because they wanted to get through it to reach the next part of the game. Hence, the game was changed to let the player exit the RT room via the map, giving them the choice to play the loop or skip it.

**Figure 5:**
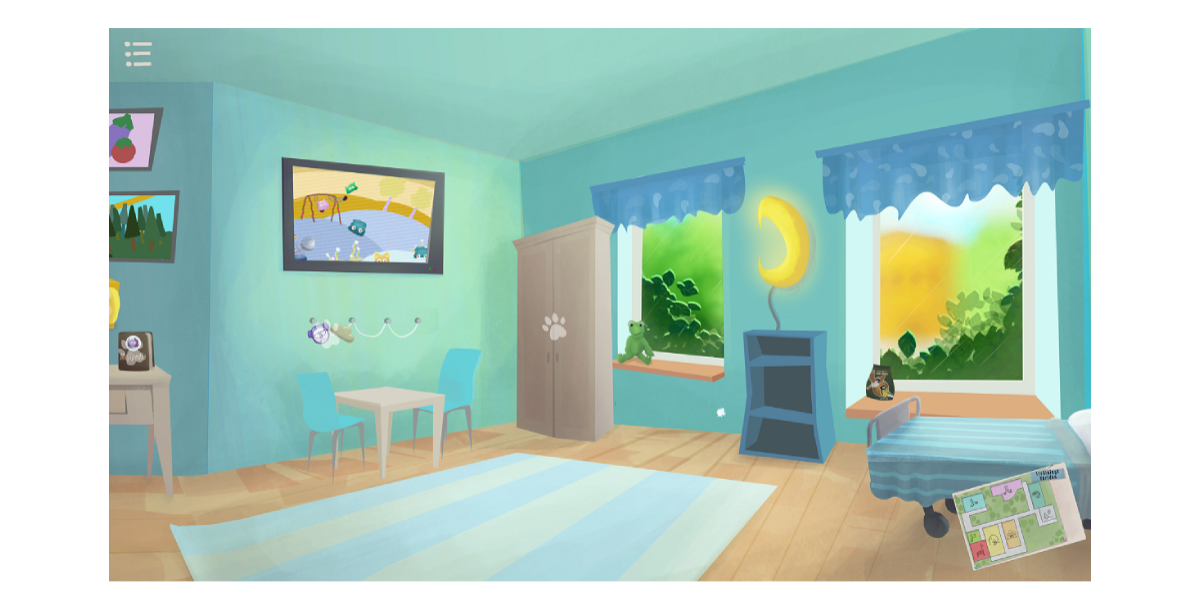
Clicking on the paw symbol on the closet reveals a hidden scene.

**Figure 6:**
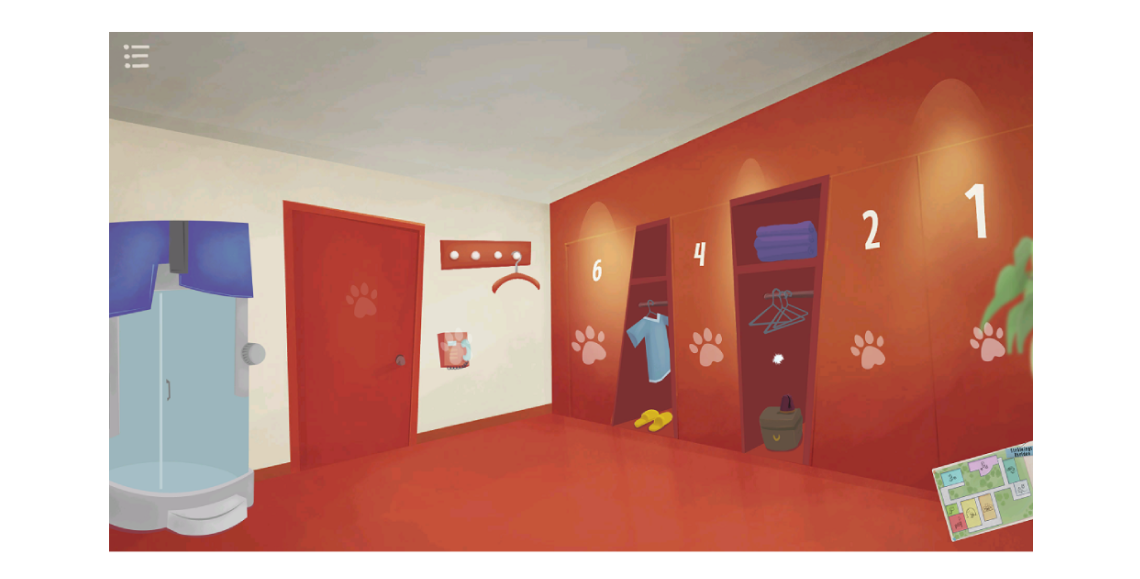
A hidden scene (a walk-in closet) revealed by the interaction in Figure 5.

A kitchen scene had a tap that started to dance and make elephant noises when the player clicked it. Two of the 7- to 8-year-old children, who were playing together, clicked on the tap several times and talked about it. One of them showed it to their parent and said, “You need to wash your hands,” and then laughed happily and called it funny. Even the older 10-year-old children laughed at the tap. Inspired by this, we added back, in random, hidden places, scenes that had previously been deleted. When the children encountered these hidden scenes, they showed them either to each other or to their parents and talked about them. When one child played the game, they commented, “This room is so cool!” explaining, “But the closet is so small, and then all of this shows up.” The interaction consisted of a changing room that was hidden within a closet (Figure 5 and Figure 6). Children who found these hidden scenes started to search other scenes more carefully.

**Figure 7:**
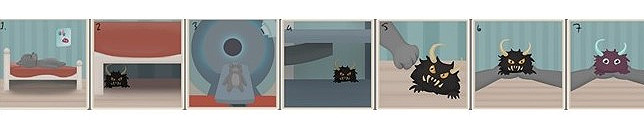
Comic strip explaining that it is normal to feel scared of the procedure, and that this feeling usually ceases after becoming accustomed to it; the monsters under the bed are an analogy.

A practice room was added to give more information about RT. One of the 7- to 8-year-old children showed particular interest in a prototype of a timer that allowed visualizing how to be still for a length of time. When asked what they were doing, the child replied, “I am practicing to see how long I can be still.” This apparent interest in the timer inspired us to portray more elements of RT in a way that would create value. This led us to develop the idea of using comic strips to explain certain elements of the RT procedure (Figure 7).

In the last round, the children showed awareness that the game had changed extensively from the first time they played it and agreed that it gave a valid introduction to RT while including elements for amusement. In all, numerous changes had been made to the game due to the children’s participation. Examples of some, but not all, of these changes are included in Multimedia Appendix 2.

### The Necessity of Understanding the Text

A significant number of the learning goals in the game were delivered through text snippets, and understanding these snippets was consequently an integral part of the player experience. Some children struggled with the amount of text; this proved most difficult for the 7- to 8-year-old children, due to their lack of reading skills. When a researcher offered to read the text, almost all the children in this age group preferred this. Reading the text gave us instant feedback about how well it worked, and allowed us to revise the text with every new version to better accommodate the 7- to 8-year-old players. The game’s final version included audio of the text that the player could activate.

We also implemented comic strips with explanatory text to increase understanding of what RT is like and what can happen when receiving RT. All these comics used metaphors for various elements of the treatment (Figure 7). The comics also used relatable, real-life analogies to describe and create an understanding of what could happen when receiving RT.

The children that read the comic strips were asked about them. Some children asked questions while reading them. These questions were often about the text; the children were sometimes confused when the text did not correspond to the picture or when the same text was used for a different picture. However, when they read the entire comic strip, they seemed to understand its overall purpose. For example, the comics used a metaphor of “monsters under the bed” that the children related to and thought could be added to the game (Figure 7).

## Discussion

### Summary

Our findings represent a thorough description of how children were part of the development of a serious game aimed at increasing knowledge about RT and decreasing anxiety related to the procedure. We performed a thematic analysis that revealed two main themes and several subthemes. The main themes were that the children’s participation was affected by their health and treatment, and that participation allowed becoming an active part of game development. The children’s participation influenced development of the game and led to changes to the game’s narrative, mechanics, and aesthetics. The game became more of a “doll-house” style experience, focusing on exploration and discovery, with the addition of a map that made it possible to move freely between the rooms [25]. With the exception of mini games, winning conditions were removed to make space for free, unstructured play. To ensure that the younger, 7- to 8-year-old children could manage to play, their input on game mechanics was prioritized, because they are more often in need of anesthesia and therefore more likely to be the end users. The participation of the 10-year-old children added knowledge about the RT procedure and the depiction of the game environment. The parents’ participation did not lead to more than a few changes, but their participation facilitated the meetings for the children.

### Principal Findings of Participation

#### Severe Illness Affects the Possibility to Participate

It proved to be crucial to modify the meetings to accommodate the children’s need for rest due to their illness and treatment, so that they could continue their participation. Discussing a meeting’s time and place with the parents and the participants prior to the meeting was a way to adjust the requirements. To include participants in the decisions on when, where, and for how long to hold the meetings enabled the participants to be part of the development of the game, as well as the design of the study [26]. This change was primarily made due to COVID-19, rather than the children’s illnesses as such, but it nevertheless facilitated participation. This finding is imperative for researchers planning to involve similar children. Children are considered to be a vulnerable group due to their stage of cognitive development; they are often regarded as not being able to fully comprehend the research in which they are taking part. The group in this study was also considered vulnerable due to the severity of their illnesses [27]. Nevertheless, it has been established that including children in the development of future interventions is necessary, because the intervention concerns them as a group [26]. However, since our group could not meet all at once, the children themselves could not reach a consensus on what changes were needed in the game; that had to be established by the lead investigator. Hence, the investigator had to search the children’s game play and interviews for commonalities and differences in the material and then create a list of proposed changes to present to the design team. This is not a common way of working in developmental design, but it might be a plausible way to enable seriously ill 7- to 11-year-old children to be part of the process. Further, when using PAR, children are not only a source of data but a part of development [28] and are partners capable of contributing to the results [29]. The children who participated in this study became both stakeholders in the product under development and also the voices of the end users. On Shier’s ladder of participation [30], the children were somewhere between “shared decisions with children” and “child initiated and directed participation.” While the design decisions were made by the research team and game designers, these decisions were highly informed by the children’s and parents’ input.

#### Children’s Contributions Led to More Comprehensible Gameplay

The game needed to be comprehensible to the children, both when it came to how and why to play and also the informational content; the game’s interactivity also relied on knowing how to control it.

The children’s constant feedback on how they thought the game worked allowed us to design the game to be suitable for them. As an example, the children influenced how the coping strategies were presented in the game. They commented on the designer’s mistakes, such as the previously mentioned visualization of hand movement when a parent tugged on a string, when in reality they had to be completely still, and pointed out other coping strategies they had used that were not displayed in the game. The children’s involvement in the design process allowed us to correct mistakes and add new coping strategies to make the game more accurate and provide end users more tools. Exaggerating motions, such as the hand moving to represent the tug of the string, is a common way of presenting this type of movement in animation [31]. However, as the children experienced, and pointed out to us, it was important to not move throughout the treatment, so this exaggeration was something they understood as a problem. As they had personal experience of RT, this issue was visible to them in a way that would be very hard for anyone else to see. While it may seem like an obvious thing, it was not obvious until they pointed it out. Thus, their expertise in being children gave the designers a unique understanding of the situation. With the children’s participation, the designers ensured that the RT process was understandable and correctly displayed from the children’s point of view.

When observing the children playing, the main investigator noticed that the children spent more time on, for example, a dancing kitchen tap than on RT-related issues. This demonstrated that the children enjoyed the random nature of the interaction, and therefore obtained value from it; this is similar to an observation made by Howard-Jones and Demetriou [32], who found that players were more likely to prefer unpredicted rewards over ones they had foreseen. This gave us the idea to add previously unused scenes as hidden objects to create surprise and implement and test the addition. It may seem unconventional to depict an important procedure, like RT, in a playful way. However, as Clark [33] pointed out, resilient families and their ill children use imagination and humor as a way to cope with difficult situations. The amusement provided by the game did not directly contribute to education, but even if the learning aspects of the game became side impressions, the players still came in contact with them and learned from them. Mader et al [34] recommend that game designers focus on entertainment and fun when designing serious games to even out the medical details. It needs to be noted that children and their parents preparing for RT are highly motivated to understand the procedure, or at least, not to be afraid of it. This creates a multi-faceted situation that is harder than usual to address with a serious game, whose function can normally be viewed as a process of convincing.

#### Learning Through Text

Using text as the means of learning in a serious game proved to have a roughly similar outcome as learning through game mechanics, as when using the latter, the player could misinterpret the message if they did not understand it correctly [35]. The use of metaphors to help children understand medical instructions in games has previously been tested, and the results show that children prefer different metaphors for practice [36]. In the current game, each medical phenomenon was explained by a single corresponding metaphor. The children said they understood the metaphors, but if the game is redesigned in the future, more metaphors might be added to further increase the children’s ability to understand.

### Limitations

Of the 14 families contacted, 9 chose to participate, including only 1 boy. While the data collected was rich, additional boys in the group could perhaps have provided additional information. One reason for the lack of boys was that at the time of inclusion, there were more girls than boys being treated with RT at the center. Two of the initial child participants left the study, one of whom said it was because they felt the game was more suited for younger children and one of whom did not give a reason. The data collection lasted for 8 months; thus, the procedure was time-consuming. However, since the children had different gaming backgrounds, they had different levels of background knowledge of computer and web games; this added richness to the way they comprehended the game under development that the investigators found more valuable than gender diversity. Further, these differences in gaming habits did not change when some of the children left the study. As suggested by Maheu-Cadotte et al [37], researchers should involve end users with varying gaming habits and use different methods to prompt their input when developing serious games. Using a qualitative method with several methods of data collection provided rich findings that strengthened the credibility of our conclusions and increased their transferability to similar areas under investigation [38]. Researchers use thematic analysis to develop themes that provide insight into the research question. It may be noted that the frequent concurrency of the ideas behind the themes in our data set has not been evaluated [39]; these data merely provide insight into individual experiences.

### Future Research

Further evaluation of the game is warranted to determine whether it can increase knowledge of the procedure and thereby decrease anxiety. Thus, we are currently inviting children between 5 and 14 who are scheduled to undergo RT to evaluate the game.

### Conclusion

Having children be part of the cocreation process through PAR resulted in several changes to the game. More importantly, the methods used here made the children active participants; therefore, this method can be used by health care researchers to develop cocreated serious games together with children. When children participate in research, the study framework needs revision throughout the process, due to unforeseen circumstances and in order to facilitate participation. It is necessary to inform the child participants that the process takes time, and that it can be revised to facilitate their participation as much as possible to avoid placing a burden on them.

## Appendix

Multimedia Appendix 1 Semistructured interview guide.

Multimedia Appendix 2 Listed changes made to the game.

## Abbreviations

PAR: participatory action research
RT: radiotherapy
