# Supplementary material for: Co-creation of a Serious Game About Radiotherapy: Participatory Action Research Study With Children Treated for Cancer
Source: JMIR Hum Factors. 2022 May 31;9(2):e34476. doi: 10.2196/34476 (PMC9198823; doi:10.2196/34476)
Supplement: Multimedia Appendix 1 [file humanfactors_v9i2e34476_app1.docx]

Appendix 1

Interview guide (translated from Swedish)

What did you think about the game today?

Was there anything you thought should be in the game that was not there?

What did you like in the game?

What was bad?

Did you see any changes since last time you played?

What did you think about them?

Also always included questions on specific things the children had done during gameplay for example: I saw you were in the kitchen and clicked the tap several times, can you explain why you did that?
