# Supplementary material for: Co-creation of a Serious Game About Radiotherapy: Participatory Action Research Study With Children Treated for Cancer
Source: JMIR Hum Factors. 2022 May 31;9(2):e34476. doi: 10.2196/34476 (PMC9198823; doi:10.2196/34476)
Supplement: Multimedia Appendix 2 [file humanfactors_v9i2e34476_app2.docx]

| **Room** | **Degree of changes made by the suggestions from the children** | **Examples of changes (not every change included in list)** |
| --- | --- | --- |
| Reception | Several | Tutorial |
|  |  | Amusement added, given the possibility to catch moji |
|  |  | Text modifications |
| RT room | Several | Explanations of equipment added |
|  |  | Possibility to leave the room without receiving treatment added |
|  |  | Certain visuals enhanced |
|  |  | Telephone added |
|  |  | Possibility to choose mask during treatment |
|  |  | Nurse’s movements dialed down |
|  |  | Mini game’s degree of difficulty modified |
|  |  | Mini game deleted (coping procedure) |
|  |  | Coping procedure of things to think about during RT added |
| Monitor room | Several | Explanations of monitors added |
|  |  | X-ray of body, cancer tumor added |
|  |  | Sound effects modified |
| Narcosis room | Minor | Video of character under narcosis slowed down |
|  |  | Moji removed |
|  |  | Text modifications |
| Training room | Room not pre-planned, was built to accommodate the children’s demand for more information related to RT | Objects moved for comprehensivity |
|  |  | Comic strips added |
|  |  | Text modifications |
|  |  | Bug fixes |
| Kitchen | Minor | Text modifications |
|  |  | Visual of bird seed made clearer |
|  |  | Bug fixes |
| Bedroom | Several | Hidden scenes added |
|  |  | Book about RT added |
|  |  | Mini game of rocket changed |
|  |  | Bug fixes |
